# Supplementary material for: The Role of CTLA-4 in T Cell Exhaustion in Chronic Hepatitis B Virus Infection
Source: Viruses. 2023 May 10;15(5):1141. doi: 10.3390/v15051141 (PMC10223466; doi:10.3390/v15051141)
Supplement: Supplementary file 1 [file viruses-15-01141-s001.zip › Supplementary, Table S1, Search strategy, Table S2, Table S3, and Table S4.pdf]

# Supplementary

**Table S1.** Search table for the review question: "which role the CTLA-4 receptor plays in the exhaustion state in T-cells seen in patients chronically infected with HBV".

| | **AND** | |
|---|---|---|
| **OR** | **CTLA-4** | **Hepatitis B** |
| | CTLA-4 antigen (MeSH) | Hepatitis B (MeSH) |
| | CTLA-4 (Text word) | Hepatitis B, chronic (MeSH) |
| | Cytotoxic-lymphocyte-associated antigen 4 (Text word) | Hepatitis B virus infection (Text word) |
| | CD152 (Text word) | Chronic hepatitis B (Text word) |
| | | Chronic HBV (Text word) |
| | | Persistent HBV infection (Text word) |
| | | CHB patients (Text word) |
| | | Hepatitis B (Text word) |

**Search Pubmed:**

**(("CTLA-4 Antigen"[Mesh Terms] OR ((((Cytotoxic T lymphocyte-associated antigen-4[Text Word]) OR (CTLA-4[Text Word])) OR (CD152[Text Word])))) AND ((("Hepatitis B"[Mesh Terms]) OR "Hepatitis B, Chronic"[Mesh Terms]) OR ((((((Hepatitis B Virus Infection[Text Word]) OR (Chronic Hepatitis B[Text Word])) OR (Chronic HBV[Text Word])) OR (persistent HBV infection[Text Word])) OR (CHB patients[Text Word])) OR (Hepatitis B[Text Word])))**

**Search Embase:**
**Link to search on OVID:**
https://ep.fjernadgang.kb.dk/login?url=http://ovidsp.ovid.com/ovidweb.cgi?T=JS&NEWS=N&PAGE=main&SHAREDSEARCHID=4NVOLjOOznE3eZvkzlbA7WkkUUsY1kq0brTx0wmv7b5RDoK0AVsP54KIZvV6nKFTD

1. exp cytotoxic T lymphocyte antigen 4/
2. CTLA-4.mp. [mp=title, abstract, heading word, drug trade name, original title, device manufacturer, drug manufacturer, device trade name, keyword, floating subheading word, candidate term word]
3. Cytotoxic-lymphocyte-associated antigen 4.mp. [mp=title, abstract, heading word, drug trade name, original title, device manufacturer, drug manufacturer, device trade name, keyword, floating subheading word, candidate term word]
4. CD152.mp. [mp=title, abstract, heading word, drug trade name, original title, device manufacturer, drug manufacturer, device trade name, keyword, floating subheading word, candidate term word]
5. exp Hepatitis B virus/ or exp hepatitis B/
6. Hepatitis B, chronic.mp. or exp chronic hepatitis B/
7. Hepatitis B virus infection.mp. [mp=title, abstract, heading word, drug trade name, original title, device manufacturer, drug manufacturer, device trade name, keyword, floating subheading word, candidate term word]
8. Chronic HBV.mp. [mp=title, abstract, heading word, drug trade name, original title, device manufacturer, drug manufacturer, device trade name, keyword, floating subheading word, candidate term word]

9. Persistent HBV infection.mp. [mp=title, abstract, heading word, drug trade name, original title, device manufacturer, drug manufacturer, device trade name, keyword, floating subheading word, candidate term word]

10. CHB patient.mp. [mp=title, abstract, heading word, drug trade name, original title, device manufacturer, drug manufacturer, device trade name, keyword, floating subheading word, candidate term word]

11. CHB.mp. [mp=title, abstract, heading word, drug trade name, original title, device manufacturer, drug manufacturer, device trade name, keyword, floating subheading word, candidate term word]

12. 1 or 2 or 3 or 4

13. 5 or 6 or 7 or 8 or 9 or 10 or 11

14. 12 and 1

**Table S2.** Overview of included studies. CD8+ T-cells

| Source | Aim | Study participants | Inclusion criteria | Exclusion criteria | Main T-cell type | Global - or HBV specific T cells | Primary findings |
|---|---|---|---|---|---|---|---|
| Bengsch, B, [21] (2014) | To investigate upregulation of inhibitory receptors on HBV-specific CD8+ T-cells and effect of PD-1 blockade | Total CHB: n= 98<br><br>HBV-specific CD8+ T-cell responses: n = 22<br><br>CHB liver biopsy: n = 6 | HBsAg-positive<br>HLA-A*02 positive | Antiviral therapy<br>HCV or HDV infection<br>HCC<br>Patients without full virological data | CD8+ | HBV specific | PD-1 was the primary inhibitory receptor on HBV-specific CD8+ from peripheral blood and liver biopsies. CTLA-4 expression was significantly weaker (21.4%)<br><br>Response to inhibitory receptor blockade was heterogeneous. The strongest increase in proliferation was observed in PD-L1 blockade (184%), while CTLA-4 blockade was less effective (53%). |
| Jiang, D. [22] (2022) | To investigate the phenotypic heterogeneity of exhausted CD8+ T cells in HBV | CHB: n = 31<br><br>HC: n = 23 | HBsAg positive for > 6 months<br>Normal liver function | HAV, HCV, HDV infection<br>Normal liver function<br>Other severe or active diseases | CD8+ | Global | CD8+ T cells exhibited higher levels of inhibitory receptors (CTLA-4, LAG3, TIM3 and PD1) in CHB patients than in HCs, but only PD1 and TIM3 were significantly increased<br><br>mRNA expression of CTLA4 was increased in the PBMCs of CHB patients<br><br>CTLA4 expression was slightly elevated in CXCR5+CD8+ T cells compared to CXCR5−CD8+ T cells<br><br>CD8+ T cells produced lower levels of cytokines (IFN-$\gamma$, TNF-$\alpha$, and Granzyme B). |

| | | | | | | | |
|---|---|---|---|---|---|---|---|
| Park, J. J. [23] (2016) | To examine whether T-cell effector and regulatory responses can define clinical stages of CHB | CHB: n = 200<br><br>HC: n = 20 | HBsAg-positive<br>≥ 18 years | Antiviral therapy<br>HIV infection<br>HCC<br>Hepatic decompensation<br>Liver transplant<br>Active autoimmune disease<br>Medications, or comorbid illnesses that can impact immune response. | CD8+ and CD4+ | HBV specific<br><br>(+ Flu specific) | Antiviral T-cell responses do not provide distinct immune signatures for CHB phenotypes.<br><br>No difference in level of CTLA-4 and PD-1 expression in CD8+T-cells in CHB patients compared to HC. |
| Peng, G., Luo, B. et al [24] (2011) | To characterize the association between persistent HBeAg and the properties of HBV- specific CD8 T cells, as well as the levels of liver injury in CHB patients with different HBeAg statuses. | CHB: n = 103<br><br>HC: n = 30 | CHB diagnosis<br>HLA-A2 positive | Antiviral - or steroid therapy past 6 months<br>HCV, HDV, or HIV infection<br>Other markers of autoimmune hepatitis and drug-induced hepatitis. | CD8+ | HBV specific | Differences in HBV-pentamer+ T cell frequency were not significant, but increased CTLA-4 (and PD-1 expression) on HBV-specific CD8 T cells was seen in the HBeAg+ group.<br><br>HBV-peptide stimulation with anti-CTLA-4 and anti-PD-L1 significantly increased the proliferation in PBMCs, but enhanced IFN-$\gamma$ production only in the HBeAg+ patients. |
| Schurich, A [26] (2011) | Examine the propensity of CD8+ T-cells from patients with CHB to up- | CHB: n = 86<br><br>RHB: n = 3<br><br>HC = 23 | CHB diagnosis<br>Treatment-naïve at recruitment<br>CMV seropositive | HCV or HIV infection | CD8+ | Both | CD8+ T-Cells in CHB had an enhanced propensity to up-regulate CTLA-4.<br><br>Correlation between HBV DNA and CTLA-4 expression on global CD8+ T cells. |

| | | | | | | | |
|---|---|---|---|---|---|---|---|
| | regulate CTLA-4. | | | | | | The level of CTLA-4 expression on HBV-specific CD8+ T cells was increased compared to the total CD8+ T cell population<br><br>Correlation between increased CTLA-4 and Bim on HBV-specific CD8+ T-cells. |
| Tang, ZS [28] (2016) | To investigate the expression characteristics of CD28 family on T-cells in patients with CHB and to investigate the effects of PD-1 blockade | CHB: n = 52<br><br>HC: n = 26 | HBsAg-positive | Antiviral - or immune-suppressant therapy past 1 year<br>Other liver diseases | CD8+ and CD4+ | Global | No increased expression of CTLA-4 on CD8+ T-cells compared to HC.<br><br>No correlation between viral load and increased expression of CTLA-4 on CD8+ T-cells. |
| Wang, X [9] (2019) | To explore the genetic and phenotypic difference in CD8+ T-cell exhaustion between CHB patients and HCC. | CHB/HCC: 40 (liver tissue)<br><br>HC: n = 40 (PBMCs) | NA | NA | CD8+ | Global | Increased expression of CTLA-4, PD-1, Tim-3, LAG-3 in<br>CHB and HCC liver tissue<br><br>Reduced CD8+ T-cell function and reduced production of IL-2, IFN-γ and TNF-α in CHB and HCC samples.<br><br>CD8+ T cell exhaustion existed in both CHB and HCC, but the phenotypes, functional states and underlying mechanisms are different between the two. |

| | | | | | | | |
|---|---|---|---|---|---|---|---|
| Yu, Y [11] (2009) | To test the efficacy of RNAi against CTLA4 in human PBMCs of CHB patients and to determine whether there was any difference in the Th-responses after siRNA transfection. | CHB: n = 120 | HBsAg + anti-HBcAb- positive for > 6 months | Antiviral - or immune-suppressive therapy<br>HCV, HDV or HIV infection<br>History of alcoholism<br>Patients who did not participate in all phases of the study protocol | Lympho-cytes (PBMCs) | Global | A significant positive correlation between CTLA4 mRNA and HBV DNA mRNA levels.<br><br>siRNAs could downregulate the mRNA expression of CTLA4.<br><br>The expressions of IFN-γ and IL-2 were upregulated in lymphocytes transfected with siRNA-1. |

CHB = chronic hepatitis B virus infection; CHB pre = pre-treatment; CHB post = post treatment with antiviral therapy; HC = healthy controls; RHB = resolved HBV infection; HCC = hepatocellular carcinoma; HIV = human immunodeficiency virus; HCV = hepatitis C virus; HDV hepatitis D virus; CMV = cytomegalovirus; NA = nucleos(t)ide analogs; PBMC = peripheral blood mononuclear cells; seq-IFN = sequential interferon therapy; VL = viral load

**Table S3.** Overview of included studies. CD4+ T-cells

| Source | Aim | Study participants (n) | Inclusion criteria | Exclusion criteria | Main T-cell type | Global - or HBV specific T cells | Primary findings* |
|---|---|---|---|---|---|---|---|
| Park, J. J. [23] (2016) | To examine whether T-cell effector and regulatory responses can define clinical stages of CHB | CHB: n = 200<br>HC: n = 20 | HBsAg-positive<br>≥ 18 years | Antiviral therapy<br>HIV infection<br>HCC<br>Hepatic decompensation<br>Liver transplant<br>Autoimmune disease<br>Medications, or comorbid illnesses that can impact immune response | CD4+ and CD8+ | HBV specific<br><br>(+ Flu specific) | CTLA-4 expression levels in CD4+ T-cells were greater in CHB patients than in HC |
| Raziourrouh, B. [25] (2014) | To characterize the memory and inhibitory phenotype of virus-specific CD4+ T- cells during CHB and the functional impact of negative regulatory molecules | CHB: n = 66<br>AHB: n = 41<br>RHB: n = 5<br>HC: n = 5 | HBsAg + anti-HBcAb-positive<br>> 6 months | HCV, HDV or HIV infection | CD4+ | HBV specific | CD4+ T-cells most frequently expressed PD-1 (77.9%) in contrast to CTLA-4 (19.6%)<br><br>CTLA-4 blockade did not increase proliferation of HBV-specific CD4+ T-cells. |
| Tang, ZS [28] (2016) | To investigate the expression | CHB: n = 52<br>HC: n = 26 | HBsAg-positive | Antiviral or immunosuppressan | CD4+ and CD8+ | Global | Levels of CTLA-4 on CD4+ T-cells were increased in CHB patients |

| | | | | | | | |
|---|---|---|---|---|---|---|---|
| | characteristics of CD28 family on T-cells in patients with CHB and to investigate the effects of PD-1 blockade | | | t therapy the past 1 year.<br>Other chronic liver diseases | | | No correlations between virological parameters and the abnormal expression of the CD28 family receptors on CD4+T-cells<br><br>Following anti-PD-L1 exposure, the expression levels of CD28, ICOS, PD-1 and CTLA-4 were increased in the CD4+ T-cells |
| Wang, L. [29] (2014) | To investigate the correlation between the expression levels of costimulatory molecules and the different states of CHB infection, including expression levels before and following antiviral treatment. | CHB pre: n = 30<br>CHB post: n = 32<br>HC: n = 30 | HBsAg-positive | HCV, HDV or HIV infection<br>Other causes of chronic liver damage | CD4+ | Global | Level of CD4+ significantly decreased in CHB patients compared to HC<br><br>The expression levels of CTLA-4 on CD4+ T-cells were significantly lower in the two CHB groups compared to HC<br><br>No correlation between viral load and CTLA-4 expression levels. |
| Wen, C. [26] (2023) | To investigate the role of CD4+CXCR5−FOXP+ T cells with CTLA4 expression in patients with CHB | CHB (treatment naïve): n = 106<br><br>HC: n = 25 | CHB | HAV, HCV, HDV, HEV or HIV infection<br>Autoimmune diseases<br>Other severe or active disease | CD4+ | Global | No significant difference in CTLA4 expression in CXCR5−FOXP3+ cells compared to CXCR5+FOXP3+ cells .<br><br>Comparable CTLA4 expression levels were observed in circulating and splenic CD4+CXCR5−FOXP3+ T cells. |

| | | | | | | | |
|---|---|---|---|---|---|---|---|
| | | HBV-related hepatic failure: n = 13<br><br>HBeAg+ CHB in telbivudine longitudinal trial: n = 15 | | | | | The expression levels of inhibitory markers in the CXCR5−FOXP3+ T population, including CTLA-4, were significantly upregulated after being stimulated by recombinant HBeAg and - HBcAg, but not HBsAg<br><br>The frequency of CD4+CXCR5-FOXP3+ T cells was significantly lower in patients with a complete response** than in non-complete responders<br><br>Substantial decrease in CTLA-4 expression at week 12 was observed in complete responders compared to non-complete responders. |

AHB = acute hepatitis B infection; ASC = asymptomatic carrier; CHB = chronic hepatitis B virus infection; CHB pre = pre-treatment; CHB post = post treatment with antiviral therapy; CMV = cytomegalovirus; HC = healthy controls; HCV = hepatitis C virus; HCC = hepatocellular carcinoma; HDV hepatitis D virus; human immunodeficiency virus = HIV; pre = pre-treatment; post = post-treatment; RHB = resolved hepatitis B infection.
*) Related to CTLA-4
**) i.e., HBeAg seroconversion and HBV DNA levels < 300 copies/ml

**Table S4.** Overview of included studies. Tregs

| Source | Aim | Study participants (n) | Inclusion criteria | Exclusion criteria | Main T-cell type | Global – or HBV specific T cells | Primary findings |
|---|---|---|---|---|---|---|---|
| Park, J. J. [23] (2016) | To examine whether T-cell effector and regulatory responses can define clinical stages of CHB | CHB: n = 200<br><br>HC: n = 20 | HBsAg-positive<br>≥ 18 years | Antiviral therapy<br>HIV-infection<br>HCC<br>Hepatic decompensation<br>Liver transplant<br>Autoimmune disease<br>Medications, or comorbid illnesses impacting immune response. | CD4+ Tregs and CD8+ | HBV specific<br><br>(+ Flu specific) | Upregulation of CTLA-4 on Tregs |
| Peng, G. [15] (2008) | To analyse the frequency and phenotype of Tregs in patients of different HBV infection status and investigate the effect of Tregs on antiviral immune responses in CHB patients. | CHB: n = 79<br><br>ASCs: n = 26<br><br>AHB: n = 12<br><br>HC: n = 20 | HBsAg-positive | Antiviral therapy or steroids past 6 months | Tregs | | No significant difference of the total Treg-frequency between groups. The frequency of Tregs was significantly increased in HBeAg+ CHB patients.<br><br>In CHB patients, the frequency of Tregs positively correlated with viral load, and the Tregs could suppress the proliferation and IFN-$\gamma$ production.<br><br>Compared with $CD25^{-}$ T-cells, $CD25^{high}$ cells had significantly elevated expression of CTLA-4. |
| Stoop, J. N., [17] (2005) | To determine whether Tregs are | CHB: n = 50<br><br>RHB: n = 9 | CHB patients | Antiviral or immuno-modulatory therapy past 6 months | Tregs | | Patients with CHB have an increased proportion of Treg compared to HC. |

| | | | | | | | |
|---|---|---|---|---|---|---|---|
| | involved in the inadequate immune response leading to CHB. | HC: n = 23 | | HIV, HAV, HCV, HDV or other viral hepatitis infection<br>Immunocompromised<br>Pregnancy | | | No correlation between viral load or hepatic inflammation and the percentage of peripheral blood Treg<br><br>Depletion of CD25+ cells from PBMCs of CHB patients resulted in an enhanced proliferation after stimulation with HBV core antigen. |
| Stoop, J.N., [27] (2008) | To examine the phenotype of FoxP3+ regulatory T cells in the liver of patients with CHB | CHB: n = 32<br><br>(7 samples used for detailed phenotypic analysis of Treg) | CHB patients | HAV, HCV, HDV or HIV infection<br>Resolved viral hepatitis | Tregs | | The liver contained a population of CD4+FoxP3+ cells that did not express CD25, while these cells were absent from peripheral blood.<br><br>Intrahepatic CD25-FoxP3+CD4+ Tcells had lower expression of CTLA-4 and HLA-DR compared to their CD25+ counterparts.<br><br>No correlation between ALT, metavir score and the proportion of Treg<br><br>Patients with a high viral load have a higher proportion of Treg in the liver, but not in blood |
| Zhang, H. H [16] (2010) | To investigate frequency and phenotype of Tregs in CHB and HCC | CHB: n = 15<br>HCC: n = 49<br>HC: n = 25 | CHB patients: CHB diagnosis | Antiviral therapy past 6 months<br>Liver cirrhosis | Tregs | Global | CHB patients had a higher percentage of circulating and liver-resident Tregs compared to HC.<br><br>In co-cultures, human hepatoma cell lines (HepG2 and HepG2.2.15) increased the expansion of Tregs – especially HepG2.2.15 |

| | | | | | | | |
|---|---|---|---|---|---|---|---|
| | | | | | | | CTLA-4 was upregulated after being co-cultured with HepG2 and especially HepG2.2.15 cells |

AHB = acute hepatitis B infection; ASCs = asymptomatic HBV carriers; CHB = chronic hepatitis B virus infection; CMV = cytomegalovirus; HAV = hepatitis A virus; HC = healthy controls; HCC = hepatocellular carcinoma; HCV = hepatitis C virus; HDV hepatitis D virus; human immunodeficiency virus = HIV; RHB = resolved hepatitis B infection.
